# Alcohol promotes breast cancer cell invasion by regulating the Nm23-ITGA5 pathway

**DOI:** 10.1186/1756-9966-30-75

**Published:** 2011-08-12

**Authors:** Amy W Wong, Qiwei X Paulson, Jina Hong, Renee E Stubbins, Karen Poh, Emily Schrader, Nomeli P Nunez

**Affiliations:** 1 Institute for Cell and Molecular Biology, University of Texas, Austin, TX, USA; 2 Department of Nutritional Sciences, University of Texas, Austin, TX, USA; 3 College of Natural Sciences, University of Texas, Austin, TX, USA

**Keywords:** Breast cancer, invasion, metastasis, alcohol, Nm23, ITGA5

## Abstract

**Background:**

Alcohol consumption is an established risk factor for breast cancer metastasis. Yet, the mechanism by which alcohol promotes breast cancer metastases is unknown. The ability of cancer cells to invade through tissue barriers (such as basement membrane and interstitial stroma) is an essential step towards establishing cancer metastasis. In the present study, we identify and examine the roles of two genes, *Nm23 *and *ITGA5*, in alcohol-induced breast cancer cell invasion.

**Methods:**

Human breast cancer T47D cells were treated with ethanol at various concentrations. Boyden chamber invasion assays were used to measure cellular invasive ability. The mRNA expression level of metastasis suppressor genes including *Nm23 *was determined by qRT-PCR. *ITGA5 *was identified using a qRT-PCR array of 84 genes important for cell-cell and cell-extracellular matrix interactions. *Nm23 *overexpression in addition to *Nm23*- and *ITGA5 *knock-down were used to determine the role of the Nm23-ITGA5 pathway on cellular invasive ability of T47D cells. Protein expression levels were verified by Western blot.

**Results:**

Alcohol increased the invasive ability of human breast cancer T47D cells in a dose-dependent manner through the suppression of the *Nm23 *metastatic suppressor gene. In turn, *Nm23 *down-regulation increased expression of fibronectin receptor subunit *ITGA5*, which subsequently led to increased cellular invasion. Moreover, *Nm23 *overexpression was effective in suppressing the effects of alcohol on cell invasion. In addition, we show that the effects of alcohol on invasion were also inhibited by knock-down of *ITGA5*.

**Conclusions:**

Our results suggest that the Nm23-ITGA5 pathway plays a critical role in alcohol-induced breast cancer cell invasion. Thus, regulation of this pathway may potentially be used to prevent the establishment of alcohol-promoted metastases in human breast cancers.

## Background

In 2010, approximately 200,000 women were diagnosed with breast cancer and 40,000 women were expected to die from this disease in the US [1]. Breast cancer is the second leading cause of cancer-related deaths among women in the US, after lung cancer [2]. Often, it is not the primary tumor that leads to the death of cancer patients but, rather, the metastases of the cancerous cells [3,4]. Breast cancer cells typically spread from the primary tumor site (the breast) to secondary sites (i.e. lungs, liver, bones, etc.) resulting in an increased likelihood of mortality [5]. The invasion of cancer cells into surrounding tissues is an initial step in tumor metastasis and requires the migration of cancer cells and their attachment to the extracellular matrix [6].

Cell culture and animal studies have previously shown that alcohol consumption increases the risk of developing breast cancer by increasing the ability of breast cancer cells to invade and metastasize [7,8]. Alcohol consumption increases breast cancer risk in a dose-dependent manner; the risk increases by 10% for each alcoholic drink consumed daily [7-9]. Thus, consumption of two daily alcoholic drinks may lead to a 20% increase in breast cancer risk [8]. A drink is defined as 12 oz of beer or 5 oz of wine [8]. Studies also show that alcohol may increase the risk of breast cancer recurrence in previously diagnosed women, which may affect their survival [10]. Therefore, in order to develop strategies for the prevention and treatment of alcohol-related breast cancers, it is essential to understand the molecular mechanisms by which alcohol promotes the invasive phenotype of the cancer cells. In this study, we show that alcohol promotes the invasive ability of human breast cancer T47D cells *in vitro *in a dose-dependent manner and show that the Nm23*-*ITGA5 pathway plays a critical role in the promotion of cancer cell invasion by alcohol.

Metastases suppressing genes encode proteins that hinder the establishment of metastases without blocking the growth of the primary tumor [11]. Two such genes are the human *Nm23 *genes (*Nm23-H1 *and *Nm23-H2*) which have been localized to chromosome 17q21 and encode 17 kDa proteins that use its nucleoside diphosphate (NDP) kinase [12], histidine kinase [13], and exonuclease activities [14] to inhibit multiple metastatic-related processes. Mutants that disrupt the NDP kinase and exonuclease functions of Nm23 still suppress metastasis to varying degrees, suggesting complex and overlapping roles in metastasis regulation [15]. In this report, we focus only on *Nm23-H1*. Overexpression of *Nm23-H1 *in tumor cells reduces tumor cell motility and invasion, promotes cellular differentiation, and inhibits anchorage-independent growth and adhesion to fibronectin, laminin, and vascular endothelial cells [16,17].

While *Nm23 *works to prevent the spread of breast cancer, *ITGA5 *produces an integral membrane protein that increases the metastasis of breast cancer cells [18]. *ITGA5 *is found on chromosome 12q11-q13 and encodes integrin alpha-5, a fibronectin receptor protein [19]. Through binding to fibronectin, an extracellular glycoprotein, ITGA5 facilitates cellular growth and migration [18,20]. Integrins associate with adaptor proteins, cytoplasmic kinases and transmembrane growth factor receptors to trigger biochemical signaling pathways [21]. Overexpression of *ITGA5 *leads to increased cellular adhesion and interaction with fibronectin, resulting in promoted tumor metastasis [18].

In the present study, we report, for the first time, the effects of alcohol on the Nm23-ITGA5 pathway and show that regulation of this pathway is important for *in vitro *cellular invasion of T47D human breast cancer cells.

## Methods

### Cell culture, transfection, and siRNA

T47D, MCF-7 and MDA-MB-231 breast cancer cells were purchased from American Type Culture Collection (Manassas, VA, USA). Cells were cultured at 37°C, 5% CO2, on 75-cm^3 ^tissue culture flasks (Becton Dickinson Labware, Franklin Lakes, NJ, USA) in Dulbecco's Modified Eagle's Medium (DMEM) supplemented with 10% inactivated fetal bovine serum (FBS) and 1% penicillin-streptomycin (Gibco, St Louis, MO, USA). The *Nm23 *siRNA, *ITGA5 *siRNA, and negative controls were purchased from Invitrogen (Carlsbad, CA, USA). *pcDNA3-Nm23-H1 *cDNA and the control vector were kindly provided by Dr. Patricia Steeg (National Cancer Institute, Bethesda, MD, USA). T47D cells were transfected with the above vectors and siRNAs using Lipofectamine 2000 (Invitrogen) following the manufacturer's instructions. Neomycin-resistant clones were isolated by growth in media containing 800 ug/ml G418 (Gibco, St Louis, MO, USA). Alcohol was added to the medium at concentrations of 0.1%, 0.2%, and 0.5% v/v ethanol. RNA and proteins were collected from the cells 48 hours post alcohol treatment.

### Invasion assay

The *in vitro *invasion studies were performed using the BD Bio-Coat Matrigel invasion assay system (Becton Dickinson Labware, Franklin Lakes, NJ, USA). To determine the ability of alcohol to affect the invasive ability of breast cancer cells, 2 × 10^5 ^T47D cells were suspended in serum-free DMEM medium containing 0.1% bovine serum albumin (BSA) and placed in the upper chamber. The bottom chamber was filled with DMEM containing 10% FBS. The FBS attracted the cancer cells and triggered their migration to the underside of the membrane. Breast cancer cells that have the ability to invade secrete factors which allow them to degrade the Matrigel (e.g., matrix metalloproteinases) and migrate through the 8 μm pores to the lower chamber of the membrane. After 24 hour incubation, the membrane of the upper chamber was cleaned with cotton swabs to remove the Matrigel and the cells that did not migrate. The membrane was fixed and stained using Diff-Quik solutions (Dade-Behring, Newark, DE). Staining of cells allows their visualization and quantification using a light microscope. Five fields of adherent cells were randomly counted in each well with a Nikon Diaphot-TMD (Atlantic Lab Equipment, Salem, MA, USA) inverted microscope at 20× magnification.

### Real-time reverse transcription PCR analysis

Total RNA was extracted using the RNeasy Mini Kit (Qiagen, Hilden, Germany) according to the manufacturer's instructions. Reverse transcription was performed with the High Capacity cDNA Reverse Transcription Kit (Applied Biosystems, Foster City, CA, USA), using 2 mg of RNA for each reaction. Primer pairs were designed using Primer3 software [22] and are shown in Table 1. Real-time PCR was performed with the SYBR GreenER qPCR kit (Invitrogen, Carlsbad, CA, USA) in the Mastercycler ep Realplex Real-time PCR thermocycler (Eppendorf, Wesseling-Berzdorf, Germany). The relative expression levels of target genes were normalized to the housekeeping gene 18S rRNA. Amplification specificity was confirmed by melting curve analysis.

**Table 1:** Primer sequences used for qRT-PCR

Gene name	Sequence
Nm23	F: 5'-ACC TGA AGG ACC GTC CAT TCT TTG C-3'
	R: 5'-GGG TGA AAC CAC AAG CCG ATC TCC T-3'
KISS1	F: 5'-ACC TGC CTC TTC TCA CCA AG-3'
	R: 5'-TAG CAG CTG GCT TCC TCT C-3'
Mkk4	F: 5'-GCA ACT TGA AAG CAC TAA ACC-3'
	R: 5'-CAT GTA TGG CCT ACA GCC AG-3'
RRM1	F: 5'-ACT AAG CAC CCT GAC TAT GCT ATC C-3'
	R: 5'-CTT CCA TCA CAT CAC TGA ACA CTT T-3'
KAI1	F: 5'-CAT GAA TCG CCC TGA GGT CAC CTA-3'
	R: 5'-GCC TGC ACC TTC TCC ATG CAG CCC-3'
BRMS1	F: 5'-ACT GAG TCA GCT GCG GTT GCG G-3'
	R: 5'-AAG ACC TGG AGC TGC CTC TGG CGT GC-3'
MMP1	F: 5'-CTG TTC AGG GAC AGA ATG TGC T-3'
	R: 5'-TCG ATA TGC TTC ACA GTT CTA GGG-3'
MMP2	F: 5'-TCA CTC CTG AGA TCT GCA AAC AG-3'
	R: 5'-TCA CAG TCC GCC AAA TGA AC-3'
MMP9	F: 5'-CCC TGG AGA CCT GAG AAC CA-3'
	R: 5'-CCA CCC GAG TGT AAC CAT AGC-3'
MMP13	F: 5'-TCC TCT TCT TGA GCT GGA CTC ATT-3'
	R: 5'-CGC TCT GCA AAC TGG AGG TC-3'
MMP14	F: 5'-TGC CTG CGT CCA TCA ACA CT-3'
	R: 5'-CAT CAA ACA CCC AAT GCT TGT C-3'
ITGA5	F: 5'-GTC GGG GGC TTC AAC TTA GAC-3'
	R: 5'-CCT GGC TGG CTG GTA TTA GC-3'
18S rRNA	F: 5'-TAC CTG GTT GAT CCT GCC AG-3'
	R: 5'-GAG CTC ACC GGG TTG GTT TTG-3'

### Western blot analysis

Cells were lysed using RIPA buffer containing 50 mM Tris (pH 7.6), 150 mM NaCl, 2 mM EDTA, 20 mM MgCl2, 1% Nonidet P40 containing protease inhibitors (1 μg/ml PMSF, 1 μg/ml aprotinin and 1 μg/ml pepstatin). Samples were incubated for 1 hour on ice with agitation and centrifuged at 12,000 × g for 20 min. Protein samples were subjected to electrophoresis on 4-12% SDS-polyacrylamide gradient gels and transferred to a PVDF membrane. Membranes were probed with anti-Nm23-H1 (BD Biosciences, San Jose, CA, USA) and anti-actin (Oncogene, Cambridge, MA, USA) antibodies. Protein-antibody complexes were detected with horseradish peroxidase-conjugated secondary antibodies (Cell Signaling Technology, Danvers, MA, USA) followed by enhanced chemiluminescence reaction. Immunoblots were quantified using ImageJ software (NIH website: http://rsbweb.nih.gov/ij/index.html).

### Real-time quantitative PCR array of 84 human extracellular matrix and adhesion molecules

Total RNA was extracted using the RNeasy Mini Kit (Qiagen, Hilden, Germany). The cDNA was prepared by reverse transcription using the RT^2 ^PCR Array First Strand kit (SA Biosciences, Frederick, MD) as recommended by the manufacturer's instructions. PCR array analysis of 84 genes related to cell-cell and cell-matrix interactions as well as human extracellular matrix and adhesion molecules (RT^2 ^Profiler™ PCR array, PAHS-013A-1, SA Biosciences, Frederick, MD, USA) was performed using the Mastercycler ep Realplex real-time PCR thermocycler (Eppendorf, Wesseling-Berzdorf, Germany). Briefly, 25 μl of PCR mixture, which contained cDNA equivalent to 1 μg RNA in SuperArray RT^2 ^qPCR Master Mix solution, was loaded in each well of the PCR array plate. PCR amplification of cDNA was performed under the following conditions: 10 min at 95°C for one cycle, 15 sec at 95°C, followed by 1 min at 60°C for 40 cycles. All mRNA Ct values for each sample [Ct (sample)] were normalized to glyceraldehyde-3-phosphate dehydrogenase [Ct (GAPDH)] in the same sample. The relative mRNA level was expressed as the value of 2^-ΔΔCt ^(sample).

### Statistics

One-way analysis of variance (ANOVA) was used to test the statistical significance of the qRT-PCR and invasion assay results (SPSS 12.0 student edition, SPSS Inc. Chicago, IL, USA). To detect statistical significance, p value was set at 0.05, and data are presented as the mean ± standard error of the mean (SEM).

## Results

### Alcohol increases the invasive ability of breast cancer cells in a dose-dependent manner

**Figure 1:**
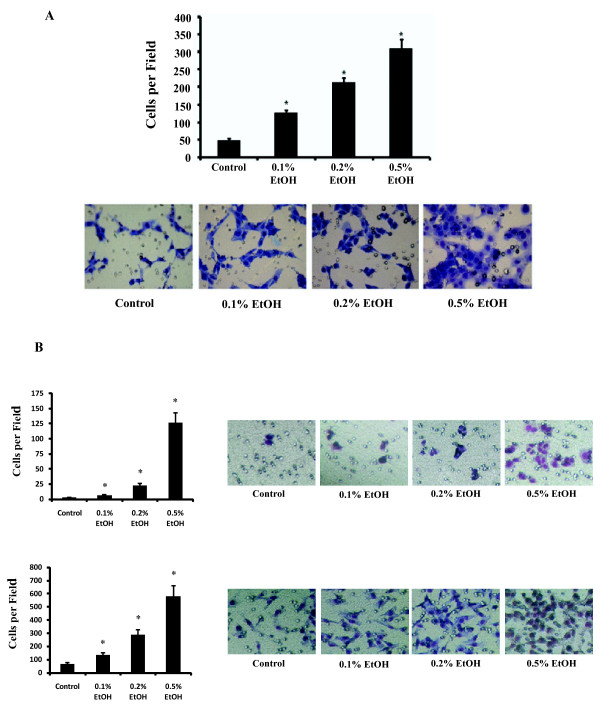
**Alcohol induces cell invasion in a dose-dependent manner**. Human breast cancer cells were treated with 0.1%, 0.2%, and 0.5% v/v ethanol for the invasion assay. (A) The top panel shows the average number of T47D cells per field that have invaded through the basement membrane-like Matrigel layer and into the lower Boyden chamber following the invasion assay. Diff-Quik staining of the lower chamber following the assay is shown below. The number of cells in the lower chamber is a direct measurement of cell invasion. (B) Invasion assay results are shown using MCF-7 (low invasive potential, top panel) and MDA-MB-231 (high invasive potential, bottom panel) breast cancer cells. (*p < 0.05, as compared to the control cells with no alcohol treatment).

To investigate the role of alcohol in cell invasive ability, human breast cancer T47D cells were treated with 0.1%, 0.2%, and 0.5% v/v ethanol for 24 hours. Previous studies have shown that alcohol exposure at these concentrations and length of time *in vitro *yielded biological effects seen in breast cancer patients [23,24]. We show that alcohol treatment *in vitro *increased the ability of T47D cells to invade in a dose-dependent manner (Figure 1A). Treatment with 0.1%, 0.2%, and 0.5% v/v alcohol increased cell invasion by approximately two-, four-, and six-fold, respectively (Figure 1A, p < 0.05). Similar results were seen with MCF-7 and MDA-MB-231, human breast cancer cell lines with low and high, respectively, invasive potential (Figure 1B).

### Alcohol increases breast cancer cell invasiveness by suppressing Nm23 expression

**Figure 2:**
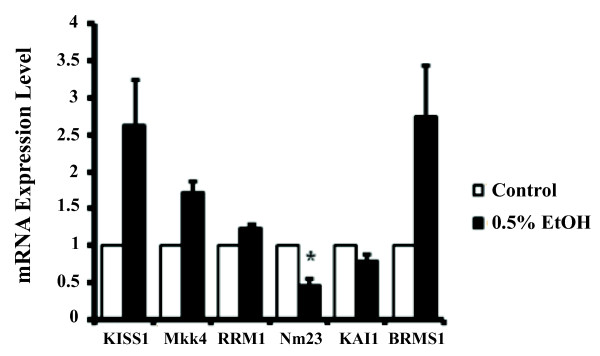
**Alcohol induces cell invasion by suppressing *Nm23 *expression**. T47D cells were treated with 0.5% v/v alcohol and the expression of known metastasis suppressor genes was determined by qRT-PCR. *Nm23 *mRNA expression levels significantly decreased following treatment. *KAI1, RRM1*, and *BRMS1 *expression were not affected by alcohol and expression of *KISS1 *and *Mkk4 *were increased by alcohol. (*p < 0.05, as compared to the control cells with no alcohol treatment).

To investigate the possibility that alcohol may increase cellular invasive ability by inhibiting the expression of specific metastasis suppressing genes, we determined the effects of alcohol on known metastasis suppressor genes. We examined the effects of 0.5% v/v ethanol on the expression levels of *Nm23, KISS1, Mkk4, RRM1, KAI1*, and *BRMS1 *metastasis suppressor genes *in vitro *by qRT-PCR (Figure 2). Our results show that alcohol significantly suppressed the expression of *Nm23 *by approximately 50% (Figure 2, p < 0.05), suggesting that the *Nm23 *metastasis suppressor gene may be involved in alcohol-induced cell invasion.

**Figure 3:**
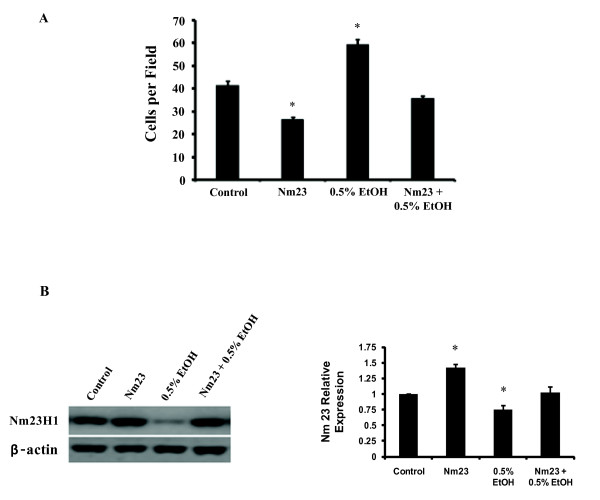
**Overexpression of *Nm23 *suppressed cell invasion**. The invasion assay was used to determine the invasive ability of T47D cells treated with 0.5% v/v ethanol and overexpressing *Nm23*, independently and in combination. (A) Alcohol treatment increased the invasiveness of the T47D cells transfected with the empty vector; however, alcohol did not increase invasion in the T47D cells transfected with *Nm23*. (B) Western blot shows Nm23 expression levels following ethanol treatment, Nm23 overexpression, and the combination of ethanol and Nm23 overexpression. Quantification by ImageJ software indicates relative Nm23 expression. (*p < 0.05, as compared to the control cells transfected with empty vector).

To determine whether the effects of alcohol on the invasive ability of T47D cells can be blocked via *Nm23*, we transfected T47D cells with the *pcDNA3-Nm23-H1 *vector (kindly provided by Dr. Patricia Steeg at the National Cancer Institute, Bethesda, MD, USA) to overexpress *Nm23*. As expected, *Nm23 *overexpression resulted in a significant decrease in T47D cell invasion (Figure 3A, p < 0.05) while treatment of T47D control cells (transfected with an empty vector) with 0.5% v/v alcohol significantly increased cell invasive ability (Figure 3A, p < 0.05). (Note: Results from Figure 1A and 3A indicate that 0.5% v/v ethanol increased cell invasion by 600% and 50%, respectively. This difference may be attributed to the addition of G418 (Gibco, St Louis, MO, USA) in the media used for the invasion assay shown in Figure 3A. As an inhibitor of protein synthesis, addition of G418 may have led to a decline in cell proliferation over the 24 hour invasion period.) However, 0.5% v/v alcohol was unable to increase the invasive ability of T47D cells overexpressing *Nm23 *(Figure 3A, p > 0.05), suggesting that *Nm23 *expression is critical in alcohol-induced T47D breast cancer cell invasion. Nm23 protein levels are shown in Figure 3B.

### Down-regulation of Nm23 increases ITGA5 expression to promote breast cancer cell invasion

**Figure 4:**
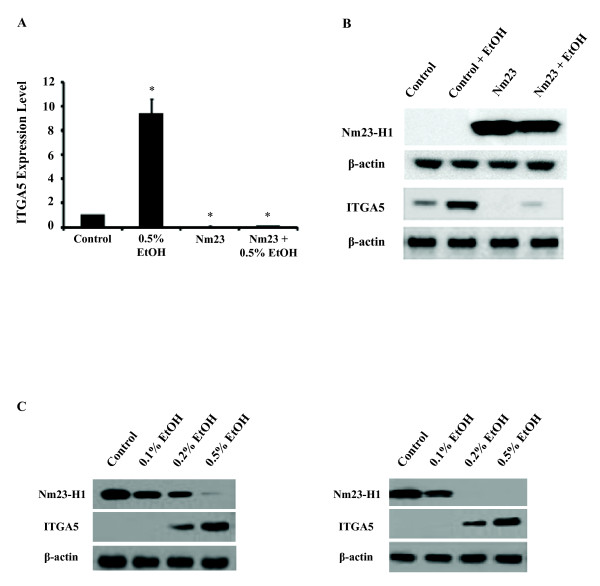
***Nm23 *down-regulates *ITGA5 *expression**. Nm23 regulates cell invasion through ITGA5 expression. (A) *ITGA5 *mRNA levels were determined by qRT-PCR in T47D cells treated with 0.5% v/v ethanol and overexpressing *Nm23*, independently and in combination. Alcohol promotes *ITGA5 *mRNA expression approximately nine-fold. This effect was blocked by the overexpression of *Nm23*. (B) Western blot shows Nm23 and ITGA5 protein level in T47D cells with ethanol treatment, Nm23 overexpression, and in combination. (C) Western blots show Nm23 and ITGA5 protein level in MCF-7 (left) and MDA-MB-231 (right) cells following various doses of ethanol treatment. (*p < 0.05, as compared to the control cells transfected with empty vector).

To examine the downstream targets of Nm23 involved in alcohol induced cell invasion, we determined the effects of *Nm23 *overexpression and 0.5% v/v ethanol treatment on 84 genes associated with extracellular matrix regulation and adhesion molecules in the following groups of breast cancer cells: 1) T47D controls cells (empty vector), 2) T47D cells treated with 0.5% v/v alcohol (empty vector), 3) T47D cells overexpressing *Nm23*, and 4) T47D cells overexpressing *Nm23 *and treated with 0.5% alcohol. Results are presented in Table 2, with only the most significantly affected genes shown. Interestingly, one gene observed to be affected by alcohol and *Nm23 *in the opposite manner was fibronectin receptor subunit integrin alpha 5 (*ITGA5*). In cells overexpressing *Nm23*, alcohol treatment was no longer able to increase *ITGA5 *expression (Table 2). Additionally, alcohol exposure increased the expression of *ITGA5 *nine-fold; however, this effect was eliminated by the overexpression of *Nm23 *(Figure 4A and Table 2), suggesting that *Nm23 *blocked the effects of alcohol. Thus, our data suggests that the effects of alcohol on *ITGA5 *are *Nm23*-dependent.

**Table 2:** Effects of alcohol and *Nm23 *overexpression on extracellular matrix and adhesion proteins expression

Gene Name	0.5% EtOH	Nm23-H1	0.5% EtOH + Nm23-H1
VCAN	4.1125	3.1514	4.359
COL8A1	-18.2522	-18.6875	-8.9755
CTGF	-4.3772	-5.712	-4.1296
CTNNA1	-15.455	-20.1681	-14.5808
CTNNB1	5.6569	5.5251	5.9134
CTNND1	-69.551	-18.9483	-26.4647
CTNND2	16.9123	12.9601	17.9262
ITGA1	-1.7777	-2.3168	-1.6771
ITGA2	-6.4531	-8.421	-6.0881
ITGA4	-5.3889	-7.0323	-5.0841
**ITGA5**	**9.3827**	**-12.0754**	**-9.038**
ITGA6	-1.1408	-1.4886	-1.0762
ITGA7	-8.1681	-7.5371	-5.4869
ITGAL	-6.3643	-8.3051	-6.0043
ITGAV	-2.042	-2.6647	-1.9265
ITGB1	-3.0314	-3.2355	-1.554
ITGB2	-2.3295	-3.0398	-2.1977
ITGB3	-5.2416	-4.8032	-3.8798
ITGB4	-1.021	1.8226	1.6066
ITGB5	-19.4271	-15.3908	-3.62
KAL1	1.454	1.1142	1.5411
LAMA1	1.1096	-1.1761	1.1761
MMP1	4.1487	-1.136	1.2176
MMP10	-12.5533	-11.3451	-5.191
MMP13	24.761	18.9746	26.2455
MMP16	4.1989	4.1583	5.6334
MMP2	3.249	1.7363	2.3685
NCAM1	-3.8106	-4.9726	-3.595
PECAM1	-13.4543	-17.5573	-12.6933
SELE	1.2483	-1.0454	1.3232
SELL	7.0128	5.374	7.4333
SELP	-7.1107	-9.2792	-6.7085
SGCE	1.021	-1.2781	1.0822
SPG7	10.4107	6.0043	8.2477
CLEC3B	-1.4641	-1.9106	-1.3813
TNC	-3.9177	-5.1124	-3.6961
VCAM1	1.0281	1.325	1.0898

**Figure 5:**
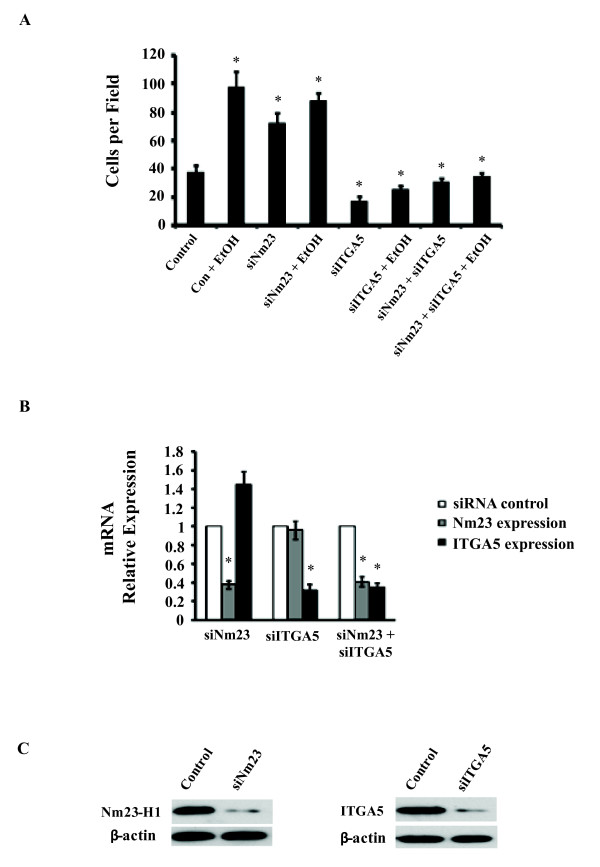
***Nm23 *knock-down promotes cell invasion and increases *ITGA5 *expression**. *Nm23 *and *ITGA5 *were knocked down via siRNA to determine their effects on T47D cell invasion. (A) The invasion assay showed that alcohol and siNm23 independently increased cell invasion. *ITGA5 *knockdown by siRNA suppressed EtOH and siNm23-induced cell invasion in T47D cells. *ITGA5 *siRNA decreased cellular invasion. (B) Following siNm23 in T47D cells, mRNA expression of *Nm23 *was reduced 62% while *ITGA5 *mRNA expression increased relative to the siRNA control. siITGA5 in T47D cells resulted in a 65% knock-down of *ITGA5 *expression and *Nm23 *levels were not affected. Double siRNA of *Nm23 *and *ITGA5 *suppressed the expression of both to less than 40%. (C) Western blot shows expression of Nm23 and ITGA5 following siRNA. (*p < 0.05, as compared to the control cells).

To determine the relationship between Nm23 and ITGA5 in alcohol-treated T47D breast cancer cells, we knocked down each gene separately and in combination, using small interfering RNA (siRNA), and subsequently measured cell invasion. If alcohol increases the invasive ability of T47D cells through the down-regulation of *Nm23*, as suggested earlier, then down-regulation of *Nm23 *should increase the invasiveness of T47D cells. Indeed, results show that knock-down of *Nm23 *by siRNA increased the invasiveness of T47D cells and alcohol was unable to further increase the invasive ability of T47D cells significantly when *Nm23 *was suppressed (Figure 5A). This work is in agreement with our results in Figure 2 and provides further evidence that alcohol increases the invasiveness of T47D cells through *Nm23*.

To establish the relationship between alcohol, Nm23, ITGA5 and cell invasion, we knocked down *ITGA5 *with siRNA in T47D cancer cells and measured the ability of alcohol to affect the invasive ability of these cells. Results show that down-regulating *ITGA5 *significantly inhibited the ability of T47D breast cancer cells to invade (Figure 5A, p < 0.05). In agreement that decreased *ITGA5 *expression reduces cell invasive ability, we show that both the *Nm23 *overexpressing cells and the alcohol-treated *Nm23 *overexpressing cells have significantly reduced *ITGA5 *expression (Figure 4A) as well as have an overall lower cell invasive ability (Figure 3A) compared to controls. We also show that alcohol-treated *Nm23 *overexpressing cells have slightly higher *ITGA5 *levels compared to non-alcohol-treated *Nm23 *overexpressing cells (Figure 4A) and this translated to a slightly higher, although not statistically significant, number of invaded cells (Figure 3A). Nm23 and ITGA5 protein expression in T47D cells is shown in Figure 4B. To examine whether the Nm23-ITGA5 effects on invasion were specific to T47D cells, we exposed MCF-7 and MDA-MB-231 cells to various doses of ethanol. We show that alcohol is able to increase Nm23 and decrease ITGA5 in a dose-dependent manner (Figure 4C) and this correlated with increasing cell invasive ability (Figure 1B). Moreover, when *ITGA5 *was knocked down with siRNA, alcohol was unable to increase the invasion of T47D cancer cells, suggesting that *ITGA5 *is necessary for alcohol to increase the invasive ability of T47D cancer cells. Furthermore, in *ITGA5 *knocked-down cells, suppression of *Nm23 *by siRNA did not rescue their invasive ability (Figure 5A). Results also show that *Nm23 *knock-down increased *ITGA5 *expression; however, knockdown of *ITGA5 *did not affect Nm23 expression (Figure 5B), suggesting that Nm23 is an upstream factor of ITGA5. Depletion of Nm23 and ITGA5 in T47D cells following siRNA transfection is shown in Figure 5C. In summary, the above findings suggest that alcohol increases the invasive ability of breast cancer cells by down-regulating *Nm23*, which increases *ITGA5 *expression, and this elevation in *ITGA5 *increases the ability of breast cancer cells to invade.

## Discussion

We show that alcohol increases the invasive ability of breast cancer cells in a dose-dependent manner. This suggests that alcohol may increase the ability of the cancer to metastasize. In fact, both animal and epidemiological findings suggest that alcohol increase the metastatic ability of breast cancers [4]. Vaeth et al. showed that frequent alcohol drinkers were 1.45-times more likely to be diagnosed with later stage breast cancer than infrequent drinkers [25]. Additionally, animal studies suggest that alcohol consumption increases the incidence of lung metastasis [26]. Thus, it is critical to understand the mechanism by which alcohol promotes the invasive ability of breast cancer cells in order to develop prevention and treatment options for cancer metastasis. Our data suggest that alcohol increases the invasive ability of breast cancer cells via the *Nm23 *metastasis suppressor gene. More importantly, we show that the invasive ability associated with alcohol can be blocked by regulating *Nm23 *levels.

The expression of integrins (e.g., ITGA5) in cancer cells is essential as they allow the cells to attach to the endothelium found within the blood vessels of organs such as the lungs (a secondary site for tumor metastasis) [27]. Thus, the levels of integrins such as ITGA5 determine how aggressively the cancer cells may spread to secondary tissues. Our data shows that alcohol exposure increases the expression of the fibronectin receptor subunit *ITGA5 *in T47D breast cancer cells. Furthermore, overexpression of *Nm23 *can block the effects of alcohol on *ITGA5 *expression. Additionally, results show that suppression of *Nm23 *by siRNA increases the expression of *ITGA5 *in the cancer cells, thus, indicating that *Nm23 *regulates *ITGA5 *expression. Furthermore, we show that down-regulation of *ITGA5 *is sufficient to block the effects of alcohol on the invasion of T47D cells. Further investigation with other breast cancer cell lines will be necessary before conclusive statements can be made regarding the involvement of the Nm23-ITGA5 pathway in alcohol-induced breast cancer cell invasiveness. Nevertheless, our results indicate that alcohol decreases the expression of *Nm23*, thereby allowing *ITGA5 *to be expressed, which in turn allows T47D breast cancer cells to obtain a more invasive phenotype.

Further investigation is also necessary to better understand how alcohol regulates Nm23 expression and how Nm23 regulates ITGA5 expression. It is well accepted that alcohol may promote breast cancer development via the estrogen signaling pathway [28]. As breast cancer cells are able to produce estrogen *in vitro*, the binding of estrogen to the estrogen receptor α (ERα) may activate downstream PI3K/Akt and MAPK/ERK pathways to promote cell migration [29,30]. In a recent study, it was reported that estrogen negatively regulates Nm23 expression *in vitro *[31]. Thus, the modulation of Nm23 expression shown in this study as a result of alcohol exposure may be mediated by estrogen levels. As a NDP kinase, Nm23 may modify cytoskeleton organization and protein trafficking, possibility through ITGA5, to promote cell migration and adhesion to the extracellular matrix (ECM). Previous studies have shown that Nm23 decreases activity of Rac1, a specific nucleotide exchange factor, through binding of Tiam1 [32,33]. Reduction of Rac1 activation induces the activity of RhoA, a component in the ITGA5-mediated cellular adhesion and migration signalling pathway [34,33]. Indeed, estrogen has been found to activate RhoA and this activity is necessary for cytoskeletal remodelling and for the enhancement of breast cancer cell migration and invasion [35]. Thus, down-regulation of Nm23 by alcohol may promote RhoA activation through estrogen regulation to favor ITGA5-mediated breast cancer progression.

The ECM and adhesion molecules play a critical role in the invasive phenotype of cancer cells [36]. For example, the binding of integrins to ECM proteins stimulates the phosphorylation of focal adhesion kinase (FAK); this activated FAK can activate signaling pathways such as PI3K, MAPK, and ERK [37]. These pathways have been shown to regulate cell adhesion, motility, invasion, and metastasis [38]. Integrins are heterodimer cell surface receptors composed of α and β subunits. The integrin α5 subunit (ITGA5) dimerizes exclusively with the β1 integrin (ITGB1) to form the classic fibronectin receptor (α5/β1 or ITGA5B1) [39]. The interaction of α5/β1 with fibronectin (FN) plays an important role in the adhesion of cancer cells to the extracellular matrix [40]. Moreover, previous studies have shown that interaction of α5/β1 with FN promotes activation of the ERK and PI3K signaling pathways, which in turn stimulates cells to invade and produce MMPs (e.g., MMP-1 MMP-9) to facilitate invasion [41]. In our studies, we show that the integrin α5 subunit expression is necessary for alcohol to increase the invasive ability of T47D breast cancer cells. It is possible that alcohol stimulates signaling pathways such as ERK and PI3K, via α5/β1, which then increases the invasive phenotype of T47D breast cancer cells. Consequently, activated integrins may facilitate the movement and metastasis of breast cancer cells. In future studies, we will determine if alcohol affects signaling pathways such as FAK, ERK, and PI3K via ITGA5 and elucidate the role of estrogen in alcohol-mediated down-regulation of Nm23.

## Conclusions

Our data suggest that alcohol increases breast cancer cell invasion by regulating the Nm23-ITGA5 pathway. Alcohol exposure in human breast cancer T47D cells down-regulated expression of the *Nm23 *metastasis suppressor gene, leading to increased expression of the *ITGA5 *fibronectin receptor subunit, and consequently induced cellular invasion *in vitro*. Results from this work suggest that modulation of the Nm23-ITGA5 pathway may be important for the prevention and treatment of human breast cancers.

## List of abbreviations

Nm23: non metastatic cells 1; ITGA5: integrin alpha 5; KISS1: KiSS-1 metastasis suppressor; Mkk4: MAP kinase kinase 4; RRM1: ribonucleotide reductase 1; KAI1: suppression of tumorigenicity 6; BRMS1: breast cancer metastasis suppressor 1; qRT-PCR: quantitative reverse-transcriptase polymerase chain reaction; siRNA: small interfering RNA; NDP: nucleoside diphosphate; DMEM: Dulbecco's Modified Eagle's Medium; FBS: fetal bovine serum; ECM: extracellular matrix; PI3K: phosphoinositide 3-kinase; Akt: serine/threonine protein kinase; MAPK: mitogen-activated protein kinase; ERK: extracellular signal-regulated kinase; FAK: focal adhesion kinase; ERα: estrogen receptor alpha.

## Competing interests

The authors declare that they have no competing interests.

## Authors' contributions

QXP and AWW designed the study, carried out most of the experiments and analyzed the data. JH performed all invasion assays. QXP drafted the original manuscript. AWW and RES equally participated in the critical review and drafting of the final manuscript. KP and ES acquired their authorship for assistance in reviewing the final draft. NPN supervised the project. All authors have read and approved the final manuscript.
